# Reactive Oxygen Species-Related Nanoparticle Toxicity in the Biomedical Field

**DOI:** 10.1186/s11671-020-03344-7

**Published:** 2020-05-20

**Authors:** Zhongjie Yu, Qi Li, Jing Wang, Yali Yu, Yin Wang, Qihui Zhou, Peifeng Li

**Affiliations:** 1 Institute for Translational Medicine, The Affiliated Hospital of Qingdao University, Qingdao University, Qingdao, 266021 China; 2 School of Basic Medicine, Qingdao University, Qingdao, 266071 China; 3 Department of Emergency Medicine, The Affiliated Hospital of Qingdao University, Qingdao University, Qingdao, 266003 China; 4 Oral Research Center, Qingdao Municipal Hospital, Qingdao, 266011 China; 5 Center for Stomatology, The Affiliated Hospital of Qingdao University, Qingdao, 266003 China

**Keywords:** Reactive oxygen species, Nanoparticles, Oxidative stress, Biotoxicity

## Abstract

The unique physicochemical characteristics of nanoparticles have recently gained increasing attention in a diverse set of applications, particularly in the biomedical field. However, concerns about the potential toxicological effects of nanoparticles remain, as they have a higher tendency to generate excessive amounts of reactive oxygen species (ROS). Due to the strong oxidation potential, the excess ROS induced by nanoparticles can result in the damage of biomolecules and organelle structures and lead to protein oxidative carbonylation, lipid peroxidation, DNA/RNA breakage, and membrane structure destruction, which further cause necrosis, apoptosis, or even mutagenesis. This review aims to give a summary of the mechanisms and responsible for ROS generation by nanoparticles at the cellular level and provide insights into the mechanics of ROS-mediated biotoxicity. We summarize the literature on nanoparticle toxicity and suggest strategies to optimize nanoparticles for biomedical applications.

## Introduction

Nanoparticles (NPs) are a class of novel synthetic particles with dimensions < 100 nm. Depending on their shape and size, the distinct physical and chemical characteristics give NPs different functions. NPs are widely used in many consumer products, including textiles, cosmetics, water purification, and food packaging [1, 2]. They are also used in the engineering of photocatalysts, energy, and optoelectronics [3–6].

In particular, NPs have become a favored material in biomedical materials and are widely used in biosensors, siRNAs delivery, targeted gene knockdown, drug delivery, and in bio-filling medical materials [7–11]. Further uses of NPs are still being discovered. For example, Duan et al. [12] showed that Fe_3_O_4_-polyethylene glycol-polyamide-amine-matrix metalloproteinase2@ chlorin e6 (Fe_3_O_4_-PEG-G5-MMP2@Ce6) nanoprobes significantly inhibited gastric tumor growth. In another case, pDNA-polyethylenimine CeO nanoparticles (pDNA-PEI-CeO NPs) could induce more fibrosarcoma cell apoptosis [13]. Furthermore, hollow silica-Fe-polyethylene glycol-human epidermal growth factor receptor 2 nanoparticles (HS-Fe-PEG-HER2 NPs) could selectively bind tumor cells and were used as imaging agents to distinguish normal tissue from cancerous cells [14]. Finally, silver nanoparticles (Ag NPs) serve as nano-antibiotics, which efficiently combat resistant bacterial biofilm-associated infections [15].

Despite the potential for positive applications of NPs in various fields, an increasing number of studies have indicated their adverse effects on organisms [16, 17] and cells following NP exposure [18, 19]. The toxic potential of NPs is dependent on their size and shape, which determined their propensity to induce the generation of reactive oxygen species (ROS) [20, 21]. The excess generation ROS may induce an array of physiopathologic outcomes, including genotoxicity, apoptosis, necrosis, inflammation, fibrosis, metaplasia, hypertrophy, and carcinogenesis [18, 22, 23]. The toxicity of NPs has also been shown to enhance the expression of pro-inflammatory cytokines and activate inflammatory cells, such as macrophages, which further increase the generation of ROS [23, 24]. The increased generation of ROS following exposure to NPs has been also shown to induce the modulation of cellular functions, with fatal results in some cases [17, 23, 25]. In this review, we discuss the main mechanisms underlying the ROS bursts induced by NPs, analyze the primary reasons for the cytotoxicity of NPs, and summarize the potential pathogenic effects of NPs. Our present review provides overwhelming evidence that the over-production of ROS is the major cause of the biotoxicity of NPs. Therefore, novel research should aim to reduce the cytotoxicity of NPs by designing NPs which induce low ROS production.

## The Application of NPs in the Biomedical Field

NPs have been used in a variety of medical applications, and several novel NPs exhibit properties which are promising for their use in novel biomedical materials. As summarized in Table 1, Nano-C60 can be used as an anticancer agent, which inhibits cancer cell proliferation, both in vivo and in vitro [26]. ZnO NPs have been used as fillers in orthopedic and dental implants [38]. TiO_2_ can be used as antibacterial agents, in air and water purification, and for dental prostheses [52–54]. Davaeifar et al. reported that a phycocyanin-ZnO nanorod could protect the cell by decreasing endogenous ROS generation [68]. Pacurari et al. pointed out that SWCNTs could be applied as a clinical diagnostic agent and as bioengineering materials [88]. Beyond that, numerous NPs can be used as antimicrobial agents, which kill bacteria by inducing ROS bursts (Table 1).

**Table 1:** NPs played their biologic role by inducing ROS burst in cells

No.	Type of NPs	Potential applications	ROS	Dose	Molecule mechanism of biotoxicity	References
1	Nano-C60	Antibacterial agents, Anticancer agents.	↑	1 μg/mL	Necrosis, apoptosis, autophagy, DNA fragmentation, cell membrane damage.	[26–28]
2	Carbon-based nanodots	Antibacterial agents.	↑	> 1 mg/mL	Oxidize the phospholipids, destroy the membranes.	[29]
3	Ag	Antibacterial agents.	↑	150 μg/mL	Intracellular oxidation, membrane potential variation, membrane permeability disruption, DNA damage, genomic instability, cell cycle arrest, cellular contents release, inactivate proteins, autophagy, disturb electron transfer process.	[30–36]
4	Gold-silver nanocage	Antibacterial agents.	↑	2.5 μg/mL	Destruction of cell membrane, apoptosis.	[37]
5	ZnO	Wastewater purification, antibacterial agents, antitumor agents, fillers in orthopedic, and dental implants.	↑	20 μg/mL	Disintegration the cell membrane, inhibition enzyme activity, inhibition DNA synthesis, DNA damage, interruption of energy transduction, mitochondrial damage, apoptosis, intracellular outflow, mitotic arrest, carcinogenic.	[38–45]
6	Gold	Anticancer agents, antibacterial agents.	↑	20 μM	Collapse membrane potential, inhibit ATPase activities, inhibit the subunit of ribosome.	[46, 47]
7	MgO	Antibacterial agents, anticancer agents.	↑	100 mg/mL	Lipid peroxidation, apoptosis.	[48, 49]
8	Fe_3_O_4_	Antibacterial agents.	↑	32 μg/mL	DNA cleavage.	[50]
9	CdSe	Antibacterial agents.	↑		Inhibition proliferation.	[51]
10	TiO_2_	Antimicrobial agents, air and water purification, dental prosthesis.	↑	10 μg/mL	Loss respiratory activity, interfere oxidative phosphorylation, DNA lesions, mitochondrial dysfunction, carcinogenicity.	[52–57]
11	Al_2_O_3_	Antibacterial agents, cross-linker.	↑	0.16 mg/L	DNA damage, mutagenesis.	[58, 59]
12	VO_2_	Antimicrobial agents.	↑	2.5 μg/mL	Mitochondrial dysfunction apoptosis.	[60, 61]
13	V_2_O_5_	Antimicrobial agents.	↑	20 mg/L	Interruption mitochondrial function.	[62, 63]
14	PCAE	Antimicrobial agents.	↑	30 μg/mL	Membrane damages.	[64]
15	Co-ZnO	Antimicrobial agents.	↑	20 μg/mL	Low toxicity.	[65]
16	Hybrid Gold/Polymer	Antimicrobial agents.	Unknown	Unknown	No cytotoxicity.	[66]
17	Ag-Fe NPs	Antimicrobial agents.	↑	100 mg/L	LDH release, disruption membrane integrity.	[67]
18	Phycocyanin-ZnO nanorod	Protect cell.	↓	50 μg/mL	Decrease in ROS production.	[68]
19	Ag/lyz-Mt	Antimicrobial agents, water disinfection.	↑	160 μg/mL	Damage cell membrane.	[69]
20	PEGylated ZnO	Antimicrobial agents, biological labeling.	↑	45 ppm	Low cytotoxicity.	[70]
21	CdS NPs	Antimicrobial agents.	↑	4 μg/mL	Inhibition proper cell septum formation, change morphology, fragment nuclei.	[71]
22	CdTe	Antimicrobial agents.	↑	0.4 mg/L	Morphological damages, apoptosis, genotoxicity.	[72]
23	ZnO@APTMS/Cu QDs	Antibacterial agents.	↑	1.4 × 10^-4^ M	Inhibition proliferation.	[73]
24	CuO	Antimicrobial agents.	↑	5 mg/L	Increase cell permeability, lipid peroxidation, DNA damage, morphological alterations, mitochondrial dysfunction, interruption ATP synthesis.	[74–76]
25	Mn_3_O_4_	Antioxidant.	↓	20 ng/μL	Protect biomolecules against ROS.	[77]
26	PEGylated nanoceria	Antioxidant.	↓	10 μM	Cell protection, radical scavenger.	[78]
27	CeO_2_	Against oxidative damage.	↓	2.5 μg/mL	Suppressed ROS production, protect cells, and tissues.	[79]
28	AuNPs-rGO-NC	Anticancer agents, antimicrobial agents.	↑	50 μg/mL	Reduction cell activity,	[80]
29	CONPs	Anticancer agents.	↑	10 μM	DNA damage.	[81]
30	Graphene	Cancer theotherapy, bioimaging, biosensing.	↑	25 μg/mL	DNA damage, mutagenesis.	[82, 83]
31	Fe_2_O_3_	Antibacterial agents.	↑	80 μg/mL	DNA damage.	[84]
32	NiO	Antibacterial agents.	↑	10 mg/L	DNA damage.	[85, 86]
33	PtAuNRs	Anticancer agents.	↓	OD at 0.5	Induce hyperthermia.	[87]
34	SWCNTs	Clinical diagnostic agent, bioengineered research.	↑	50 μg/cm^2^	DNA damage.	[88]
35	bsCdS	Anticancer agents.	↑	15 μg/mL	Apoptosis, depletion ATP, DNA damage.	[89]
36	Ag@OTV	Against H1N1 infection.	↓	Unknown	Less cytotoxicity.	[90]
37	PATA3-C4@CuS	Antibacterial agents.	↑	5.5 μg/mL	Less cytotoxicity.	[91]

## The Mechanisms of Increased ROS Induced by NPs in Cells

ROS are chemically reactive particles that contain oxygen, including hydrogen peroxide (H_2_O_2_), reactive superoxide anion radicals (O^2-^), and hydroxyl radicals (•OH) [92, 93]. ROS are predominantly generated in organelles such as the endoplasmic reticulum (ER), in peroxisomes, and most notably in the mitochondria [94]. During oxidative phosphorylation, oxygen is used for the synthesis of water by the addition of electrons through the mitochondrial electron transport chain (ETC). Some of these electrons are accepted by molecular oxygen to form O^2-^, which can further transform H_2_O_2_ and •OH [93].

In a physiological context, ROS are produced as a natural response to the normal metabolism of oxygen [95] and serve a vital role in various cellular signaling pathways [96, 97]. Dröge and Holmstrom et al. reported that ROS could activate numerous signaling cascades, including the epidermal growth factor (EGF) receptor, the mitogen-activated protein kinase (MAPK) cascades, the transcription factor activator protein-1 (AP-1), and the nuclear factor-KB (NF-κB), and further participated in the process of mammalian growth, proliferation, and differentiation [98, 99]. Further studies showed that ROS also regulated wound repair [100], survival after hypoxia [101], intracellular pH homeostasis [102], and innate immunity [103].

Nevertheless, following exposure to NPs, the intracellular generation of ROS may sharply increase by inducing ROS bursts in cells [20] (Table 1). The main mechanistic explanations for ROS bursts are that metal ions released by NPs promote ROS overexpression by impairing mitochondrial respiration [30, 104].

The metal ions released by NPs have been shown to mix into redox cycling and chemocatalysis via the Fenton reaction [H_2_O_2_ + Fe^2+^ → Fe^3+^ + HO^−^ + •OH] or Fenton-like reaction [Ag^+^ H_2_O_2_+H^+^ = Ag^+^ + •OH + H_2_O] [23, 105, 106]. The dissociated metal ion (i.e., Ag^+^) also causes cellular enzyme deactivation, membrane structure disruption [31, 107], disturbed electron-shuttling process [108], depleted redox potential levels, reduced mitochondrial membrane potentials (MMP) [109], and further enhances the accumulation of intracellular ROS. NPs have been also reported to promote the intracellular ROS accumulation by disturbing the electron transfer process [32, 110], increasing the NADP^+^/NADPH ratio [30], and interfering mitochondrial function [18]. NPs further interfere with the expression of oxidative stress-related genes, such as *soxS*, *soxR*, *oxyR*, and *ahpC* [58]; antioxidant genes, like *sod1* and *gpx 1*[111, 112]; and the NADPH production-related gene *met9* [30]. The instability in the expression of oxidative and antioxidant genes caused by NPs accelerates intracellular ROS accumulation.

**Fig. 1:**
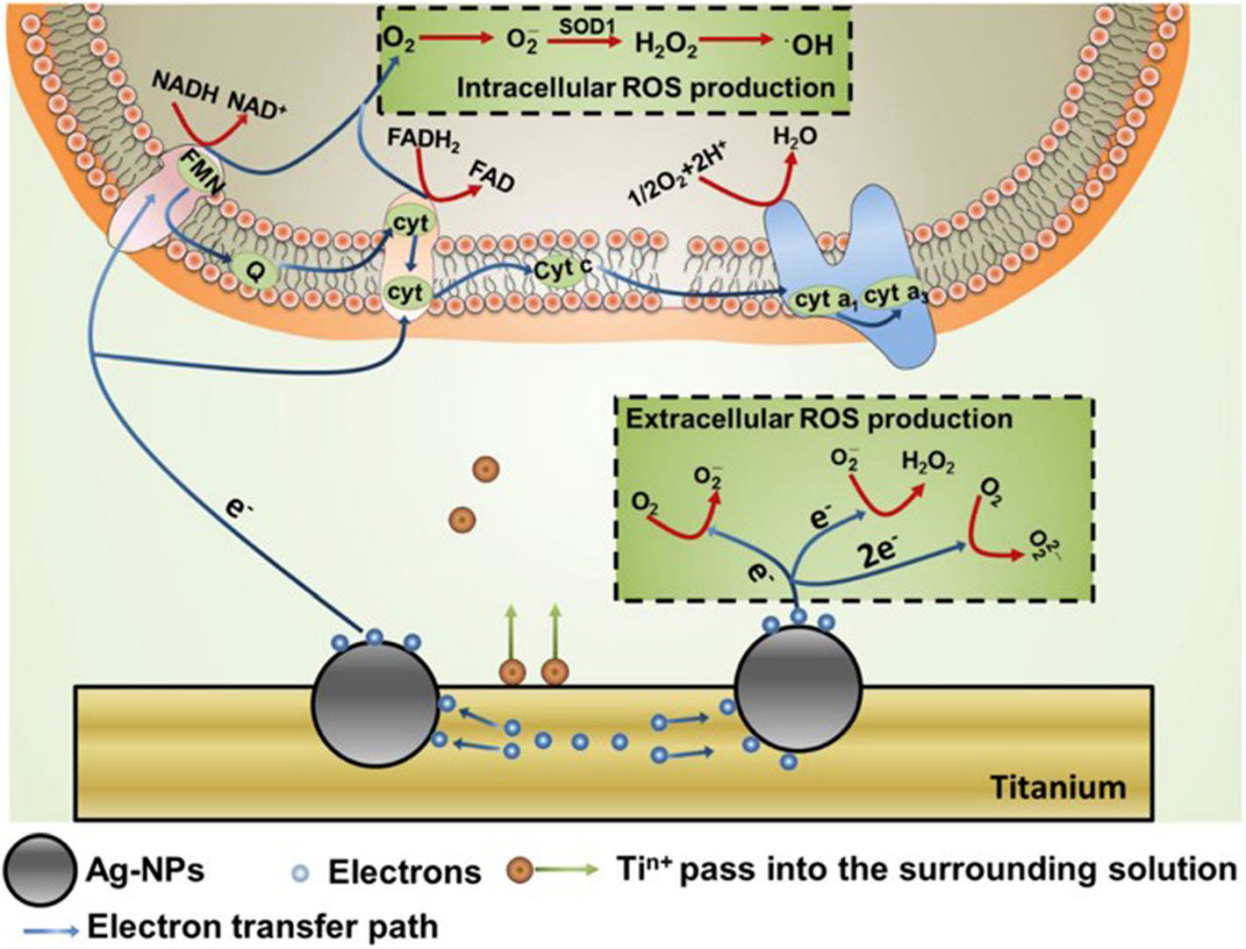
The production of ROS induced by NPs in surrounding solution and cells [32]. The electrons generated from NPs could enter into cells and disturb the functions of respiratory chain, then enhance the intracellular ROS production. Electrons also could react with O_2_ directly and increased the generation of extracellular ROS

Interestingly, increased ROS production has been strongly associated with particular sizes and shapes of NPs [113, 114]. For example, TiO_2_ NPs contributed to intracellular ROS generation, which led to nucleic acid and protein damage [10]. Liao et al. found that 10 nm TiO_2_ NPs had higher genotoxicity than other sizes tested and therefore could induce more ROS generation [115]. In another case, Se NPs promoted the production of ROS in cells, and the yield of intracellular ROS was highly associated with the diameter of Se NPs. In this case, a diameter of 81 nm induced more ROS production than other sizes tested [113]. Cho et al. further showed that the shape of NPs strongly affected their capacity to induce ROS production. Day flower-mimicking metallic nanoparticles (D-NP) lead to a significantly higher production of ROS than night flower-mimicking metallic nanoparticles (N-NP), resulting in an enhanced cell killing effect [114] (Fig. 1).

NPs can induce intracellular ROS bursts at a very low concentration (showed in Table 1), for example, Nano-C60 at 1 μg/mL can significantly increase cell apoptosis by inducing oxidative stress [26, 27]. Notably, most NPs have a dose-dependent effect, as has been reported for VO_2_ NPs [60, 61] and CuO NPs [74, 75].

## Catastrophic Consequences of NPs on Cells by Increased ROS Production

**Fig. 2:**
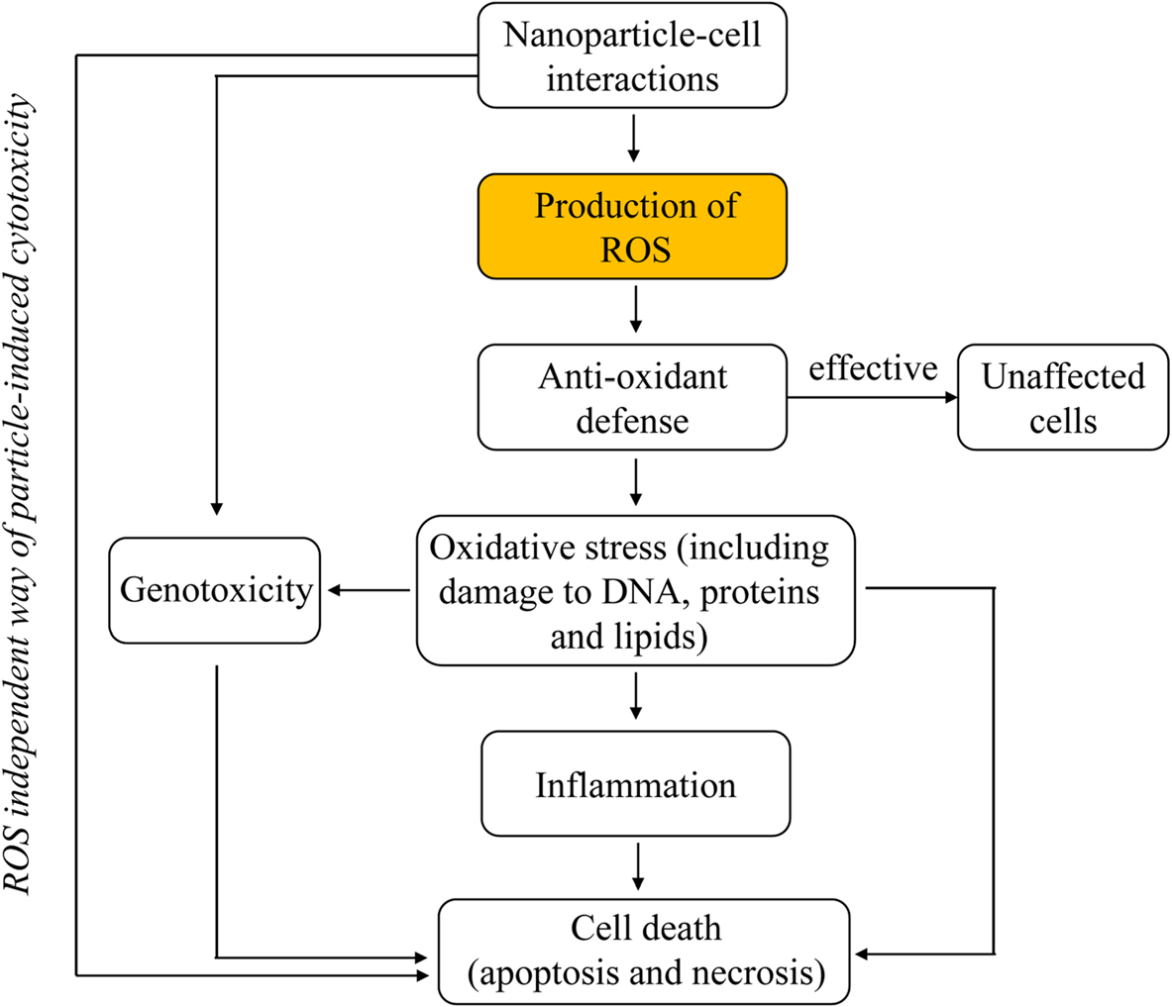
The crucial role of ROS in the cytotoxicity induced by NPs [33]. The possible cellular events taking place after NPs interact with intracellular systems

NPs which enter the cell often have adverse effects on it. The most supported explanation for the cytotoxicity of NPs is that oxidative stress is induced by a ROS burst. ROS bursts caused by NPs have resulted in the oxidative modification of biomacromolecules, in the damage of cellular structures, in the developing drug resistance, in gene mutation, and in carcinogenesis [116, 117]. Furthermore, ROS bursts have altered the normal physiological functions of cells, as in is the case with trigger inflammation, which ultimately blocks cell functions and damages the organism [23, 118, 119]. Generally, NPs are first adsorbed on the cell surface, and then passed through the membrane into the cell, where they induce ROS generation [36]. Due to its strong oxidative potential, ROS is highly stressful to cell [46] and attacks nearly all types of biomolecules in the cell, including carbohydrates, nucleic acids, unsaturated fatty acids, proteins and amino acids, and vitamins [36, 120, 121] (Fig. 2).

## ROS Results in Lipid Peroxidate and Membrane Structure Damage

Lipids, especially unsaturated fatty acids, are important intracellular macromolecules, which play key roles in the structure and functioning of the cell membrane. NPs are strongly attracted to the cell membrane, where they can generate ROS and lead to outer membrane lipid peroxidation. The altered fatty acid content of the cell membrane may result in increased cell permeability, which results in the uncontrolled transport of NPs from the extracellular environment into the cytoplasm, where cellular damage may progress further [76, 122].

Intracellular NPs induce the next round of ROS bursts. Overburdened ROS lead to the rupturing of the membranes of organelles, the leakage of the organelles’ contents [52, 123], the inactivation of cell receptors [124], the release of lactate dehydrogenase (LDH), and further irreversible cell damage [125].

### ROS Attacks Proteins and Results in Functional Inactivation

ROS attacks the hydrophobic residues of amino acids, contributing to the breakage of peptide bonds and interfering with the function of these proteins [126–128]. Carbonylation is another feature of proteins subjected to oxidative damage [129]. Carbonylated proteins form aggregates that are chemically irreversible and cannot be degraded via proteasomes, leading to the permanent loss of function in these proteins [130, 131]. Gurunathan et al. [132] showed that PtNPs could enhance the generation of ROS and increase carbonylated protein levels, which inhibited osteosarcoma proliferation and contributed to apoptosis. In one case, combustion and friction-derived nanoparticles (CFDNPs) had accumulated in the brain of young adults with Alzheimer’s disease, which likely promoted ROS generation, resulting in protein misfolding, aggregation, and fibrillation [133]. Furthermore, Pelgrift et al. showed that Mg NPs may inhibit gene transcription or damage proteins directly [10].

### ROS-Induced Gene Mutation

Nucleic acids, including DNA and RNA, are essential to cell function, growth, and development, and their component nucleotides are vulnerable targets of ROS [134–136]. Due to their low redox potential, ROS can directly react with nucleobases and modify them [137]. For example, ROS could oxidize guanine to 8-oxo-7,8 dihydroguanine (8-oxoG) [138] and adenine to 1,2-dihydro-2-oxoadenine (2-oxoA) [139]. These base modifications lead to DNA damage [140]. Because of their genotoxic potential and their capacity to induce ROS formation [141], NPs significantly induce single- and double-strand DNA breakages [142, 143], chromosome damage, and aneuploid genic events [144].

The increased production of ROS is the main cause of gene miscoding, aneuploidy, polyploidy, and the activation of mutagenesis in cells exposed to NPs [145–148]. Among the nucleotide pools, guanine is the most vulnerable and is easily oxidized to 8-oxoG by ROS [149]. The increased level of 8-oxo-dG in DNA results in the mismatch of DNA bases [150]. Similarly, the incorporation of A:8-oxoG causes an increased rate of G:C > T:A deleterious transversion mutations [151, 152]. The ratio of G:C > T:A transversion to G:C > A:T transition mutation has also been used as an index to quantify the oxidative DNA damage [153].

The generation of ROS induced by NPs resulted in the accumulation of DNA damage, which drives the development of mutagenicity [154], oncogenesis [155], multidrug resistance [156, 157], aging, and immune escape [158]. Jin et al. showed that the overproduction of ROS dramatically increased mutagenesis of DNA-binding transcriptional regulator genes, which resulted in an expedited antibiotic efflux [159], which in turn promotes the multiple-antibiotic resistance of bacteria [34]. Giannoni et al. reported that mitochondrial DNA mutations occurred with increasing intracellular ROS and further damaged the activity of ETC complex I and resulted in mitochondrial dysfunction [160, 161].

**Fig. 3:**
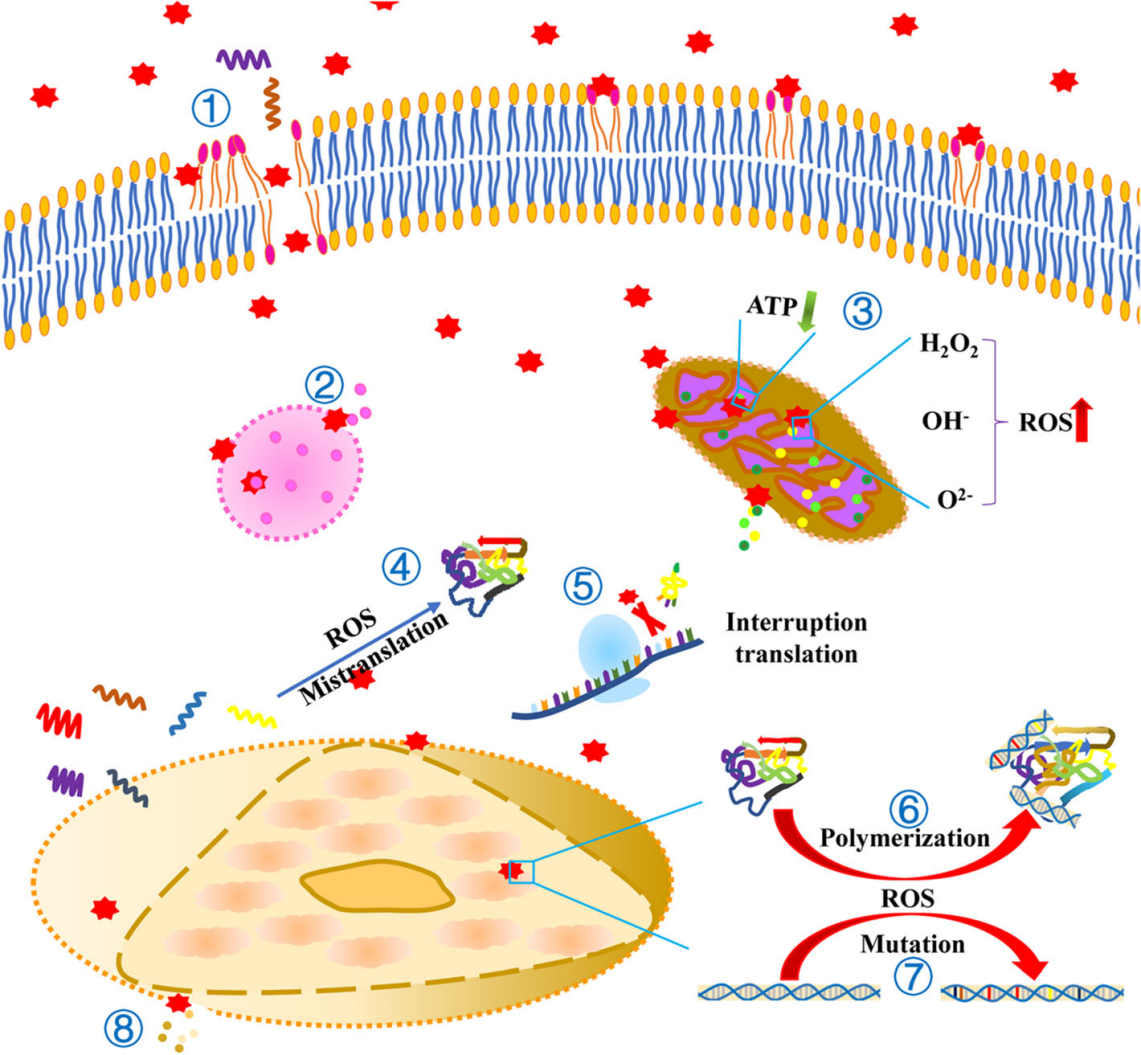
Cellular events induced by NPs. ① NPs contribute to the destruction of the cell membrane and to lipid peroxidation. ② The lysosomal membrane is destroyed by NPs and results in the release of their contents. ③ The mitochondrial membrane is damaged by NPs, leading to content release. NPs reduce the generation of ATP and increase the production of ROS. ④ The ROS induced by NPs results in the mistranslation of RNA. ⑤ NPs prevent the binding of tRNA to the ribosome. ⑥ The ROS induced by NPs result in the polymerization of proteins and DNA. ⑦ The ROS induced by NPs leads to DNA mutations ⑧ The nuclear membrane is destroyed by NPs, resulting in the release of its contents

DNA damage induced by NPs has been shown to inhibit amino acid synthesis, replication [162], and cause the aberrant accumulation of p53 [163] and Rab51 proteins [82, 142]. DNA damage may also delay or fully arrest the cell [164]. Cells with damaged DNA lose the capacity for growth and proliferation [165] and may eventually result in cell death [166] (Fig. 3).

## Increased Production of ROS Induces Cell Damage and Disease Occurrence

NP cytotoxicity is associated with oxidative stress, endogenous ROS production, and the depletion of the intracellular antioxidant pools. The increased oxidative stress leads to oxidative damage to biomacromolecules, which further affects the normal functioning of the cell and contributes to the occurrence and development of various diseases [167].

NPs induce membrane damage and enhance the transport of NPs into the cytoplasm. NPs concentrate in lysosomes, mitochondria, and the nucleus, which results in catastrophic consequences for the cell [168, 169]. It has been reported that NPs can reduce adenosine triphosphate (ATP) generation [89], deplete glutathione, induce protein mistranslation [170], rupture lysosomes [171], and inhibit the ribosomal subunit from binding transfer RNA (tRNA). These cellular events indicate a collapse of the fundamental biological process in the cell and lead to a significant decrease in cell viability [47]. Singh and Scherz-Shouval et al. reported that NPs could disturb cytoskeletal functions by inducing ROS generation and activate the process of autophagic and apoptosis in cells [89].

**Fig. 4:**
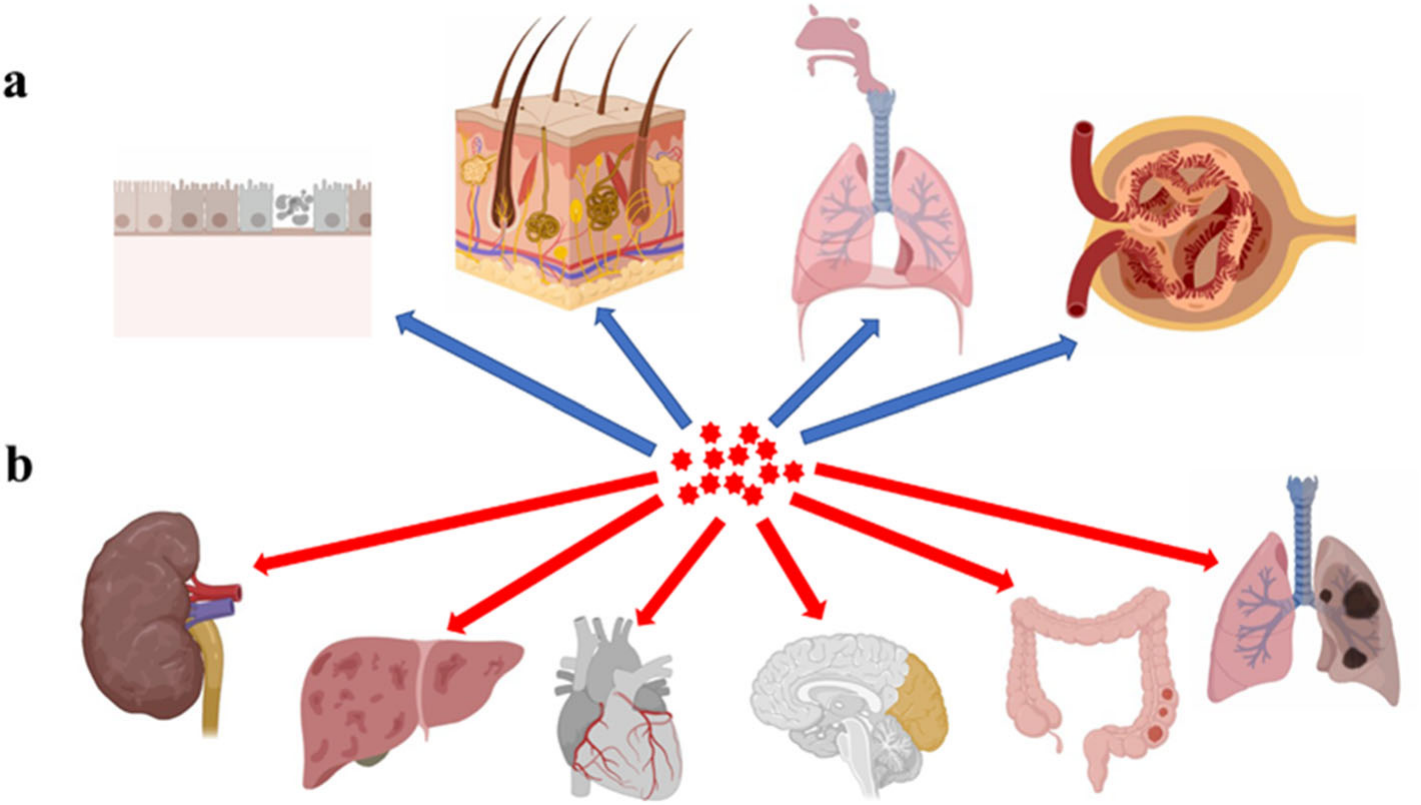
NP entrance into and damage of organs. **a** NPs could enter into the organisms through the oral cavity, nasal cavity, respiratory tract, kidneys, and intestinal tract; **b** NPs could spread by systemic circulation and accumulate in the kidneys, liver, heart, brain, intestinal tract, and lungs, leading to organ dysfunction (This figure was created in BioRender.com).

NPs enter the body via different routes, for instance through the skin, lungs, or intestinal tract (Fig. 4a) and can have a wide variety of toxicological effects and induce biological responses such as inflammation and immune responses [172–174]. In one case, exposure of cells to silica NPs caused macrophages to secrete a large amounts of interleukin-1β (IL-1β), which ultimately resulted in cell death [175]. Gao and colleagues reported that pulmonary inflammation was considerably higher in mice after exposure to carbon nanotubes, which could activate alveolar macrophages and induce a strong inflammatory response [176]. In another study, guinea pigs exposed to ZnO NPs suffered pulmonary damage, which leads to a decrease in total lung capacity and vital capacity [177–179].

ZnO NPs also induced severe injuries in the alveolar epithelial barrier and caused inflammation in the human lungs [180]. In another case, NPs absorbed into the intestines caused the inflammation and degradation of the intestinal mucosa [181]. Shubayev et al. noted that Mg NPs enhanced the migration of macrophages to the nervous system by degrading the blood-brain and blood-nerve barriers in an MMP-dependent manner [182]. Furthermore, mice which inhaled carbon nanotubes exhibited immunosuppression and repressed antibody response in naive spleen cells [183]. Finally, Cd NPs caused a severe decrease in blood monocyte viability, ultimately resulting in immunodeficiency [184].

In addition to the above pathologies, the highly variable level of ROS has been identified as the main cause of the development of numerous human diseases. Tretyakova and Liou et al. showed that oxidized DNA tends to form DNA-protein conjugates, which accumulate in the heart and brain and contribute to the occurrence of cancer, aging-related diseases, and chronic inflammation [185, 186]. Andersen [187] concluded that diabetes, as well as cardiovascular and neurodegenerative diseases, were highly related to the imbalance of ROS. Additionally, Pérez-Rosés et al. showed that increased ROS promoted Alzheimer’s and Parkinson’s disease development [188].

It has been further reported that NPs promote the apoptosis of breast cancer cells [35] and destroy malignant tissues and pathogens by promoting the generation of ROS [189, 190]. However, ROS has also been found to induce the proliferation of both normal and cancerous cells, stimulating mutations, and initiating carcinogenesis in normal cells and multidrug resistance in cancerous cells [191, 192]. Handy et al. found that fish exposed to carbon nanotubes exhibited granulomas in their lungs and tumors in their livers with extended exposure times [193]. Some NPs have caused multiple organ failure, primarily affecting the heart, lung, kidneys, and liver. TiO_2_ NPs have been shown to promote reduced body weight, spleen lesions, blood clotting in the respiratory system, necrosis and fibrosis in liver cells, and in alveolar septal incrassation [194, 195]. In one study, NPs also prevented stem cell differentiation, which aggravated organ damage [196]. Further research has also reported that NPs decreased sperm quality [197] and that exposure of sperm to carbon NPs influenced their ability to fertilize eggs and impaired the development of the embryos in purple sea urchins [198]. Mounting evidence shows the toxicological effects of NPs on microorganisms, algae, nematode, plants, animals, and humans specifically [22, 199, 200] (Fig. 4b).

## The New Type of NPs with Fewer or No Cytotoxicity

NPs possess a range of biomedical properties that make them valuable (e.g., as antibacterial and anticancer agents [26–28]). Their main mode of action is their ability to increase the production of ROS in cells; however, this property also makes these particles toxic, by causing gene mutation, apoptosis, and even carcinogenesis [45, 49, 58]. Consequently, there is an urgent need to develop new NPs which retain their required properties without leading to excessive ROS production. Recent studies have reported on novel types of NPs which could remove intracellular ROS. These types fall into two classes: (1) NPs which can scavenge ROS [77] and (2) NPs which are coated with additional materials to decrease their cytotoxicity [87].

Panikkanvalappil and colleagues showed that Pt NPs inhibit the double-strand breakage of DNA by degrading ROS [201]. In another case, Mn_3_O_4_ NPs modulated cellular redox resulting in the protection of biomacromolecules against oxidative stress [77]. Furthermore, the CeO_2_ NP is a novel agent that protects cells and tissues against oxidative damage with its free radical-scavenging capacity [79, 202].

H_2_O_2_ is the main by-product of NP-cell interactions. H_2_O_2_ destroys important biomolecules including proteins, lipids, and nucleic acids. However, when cells were treated with specialized MNPs coated with mercaptopropionic acid (MPA-NPs) or aminated silica (SiO_2_-MNPs), such damage was not observed [203, 204]. Similarly, GO coated with polyvinylpyrrolidone (PVP) has fewer toxic effects on dendritic cell (DCs), T-lymphocytes, and macrophages than without this coating. PVP-GO has been shown to reduce the apoptosis of T-lymphocytes and even increase the activity of macrophages [205]. Pt-coated AuNRs (PtAuNRs) retain the efficacy of traditional gold nanorods (AuNR) and can trigger cell death of desired cells while scavenging the ROS, thereby protecting healthy, untreated cells from the indirect death induced by ROS production [87].

## Conclusions and Outlook

NPs that possess unique physicochemical properties (e.g., ultra-small size, large surface area to mass ratio, and high reactivity) make them highly desirable in different applications. Engineered NPs for commercial purposes have been rapidly increasing. For that reason, the biosafety of NPs has gained more attention in the public. In this review, we summarized the mechanisms and responsible for ROS formation by NPs at the cellular level as well as recent advances of ROS-related NP toxicity in the biomedical field and highlighted the emerging field of cell-friendly NPs. The generation of ROS induced by NPs associated with their size, morphology, surface area, and component. In addition, ROS has bio-multifunctional in cell biology and biomedicine as well as the key mediator of cellular signaling, including cell apoptosis, viability, and differentiation.

However, to improve the biosafety of NPs and accelerate their use in the biomedical field, some bottlenecks need to be overcome and much work is still required. First, it is expected that high-throughput methods (HTMs) are designed to efficiently detect the biotoxicity of NPs in vitro and in vivo. HTMs could save time and resources, combine multiple parameters on a single system, and minimize methodological or systematic errors. It also would offer a deep understanding of the relationship between NP properties and cell responses, which could help us identify the optimal condition.

Second, the molecular and cellular mechanisms related to the biotoxicity of NP-induced ROS are still unclear. There is a demand to further explore the mechanisms associated with the formation of ROS by NPs, which would provide more information to modify the chemico-physico features of NPs to control the ROS generation. This could help researchers develop novel strategies to reduce the hazards of engineered NPs for accelerating their clinical and commercial translation in the biomedical filed.

Finally, due to their structural characteristics, NPs may enter the body freely via multiple routes, and the accumulation of NPs in the body can induce inflammation and immune responses, which result in cell injury or death, organ dysfunction, and ultimately stimulate the occurrence of numerous diseases, such as Alzheimer’s, Parkinson’s, liver inflammation, and dysembryoplasia. These issues have become more pressing with the widespread use of NPs.

## Data Availability

All data generated or analyzed during this study are included in this published article [and its supplementary information files].

## Abbreviations

•OH: Hydroxyl radical
2-oxoA: 1,2-Dihydro-2-oxoadenine
8-oxoG: 8-oxo-7,8 dihydroguanine
Ag NPs: Silver nanoparticles
AP-1: Transcription factor activator protein-1
ATP: Adenosine triphosphate
AuNR: Gold nanorods
CFDNPs: Combustion and friction-derived nanoparticles
DCs: Dendritic cells
D-NP: Day flower-mimicking metallic nanoparticles
EGF: Epidermal growth factor
ER: Endoplasmic reticulum
ETC: Mitochondrial electron transport chain
Fe_3_O_4_-PEG-G5-MMP2@Ce6: Fe_3_O_4_-polyethylene glycol-polyamide-amine-matrix metalloproteinase2@ chlorin e6
H_2_O_2_: Hydrogen peroxide
HS-Fe-PEG-HER2 NPs: Hollow silica-Fe-polyethylene glycol-human epidermal growth factor receptor 2 nanoparticles
LDH: Lactate dehydrogenase
MMP: Mitochondrial membrane potentials
MPA-NPs: MNPs coated with mercaptopropionic acid
NADP^+^/NADPH: Nicotinamide adenine dinucleotide phosphate oxidized/reduced
NF-κB: Nuclear factor-κB
N-NP: Night flower-mimicking metallic nanoparticles
NPs: Nanoparticles
O^2-^: Reactive superoxide anion radical
pDNA-PEI-CeO NPs: pDNA-polyethylenimine CeO nanoparticles
PtAuNRs: Pt-coated AuNRs
PVP: Polyvinylpyrrolidone
ROS: Reactive oxygen species
SiO_2_-MNPs: MNPs with aminated silica
tRNA: Transfer RNA
